# The Energy Absorption of a Hybridized 3D Woven Composite Under High-Velocity Impact Loading

**DOI:** 10.3390/ma18112545

**Published:** 2025-05-28

**Authors:** Kun Wang, Chao Li, Zhiming Xu, Nan Zhang, Deng’an Cai, Guangming Zhou

**Affiliations:** 1 State Key Laboratory of Mechanics and Control for Aerospace Structures, Nanjing University of Aeronautics and Astronautics, Nanjing 210016, China; wk1217@nuaa.edu.cn (K.W.); xuzhiming166@126.com (Z.X.); btycissy@nuaa.edu.cn (N.Z.); 2 Nanjing Fiberglass Research & Design Institute, Nanjing 210008, China; lichao@fiberglasschina.com

**Keywords:** three-dimensional woven composite, hybridized, high-velocity impact, energy absorption, artificial neural network

## Abstract

In this paper, the energy absorption of Kevlar fiber and carbon fiber hybridized 3D woven composites under high-velocity impact loading was studied. A high-velocity impact model was established for the composites. The 3D Hashin and von Mises failure criteria were applied for the damage criteria of the yarn and matrix, and cohesive elements were inserted between them to simulate delamination. To validate the model, simulations were compared with test results. According to the results of the model, an algorithm based on artificial neural networks was also used to predict the hybridized composites for computational efficiency considerations. In the study of optimizing the energy absorption characteristics of three-dimensional woven structures, there is an optimal position and proportion of Kevlar hybridization to ensure the stiffness index of the structure. It is found that the position of Kevlar hybridization can result in considerable enhancement in the energy absorption of the target plate in the 3D woven structure. The proportion of Kevlar content affects the energy absorption of the optimal hybrid combination of the target plate. The energy absorption of the target plate can be effectively increased by adjusting the hybrid combination of different yarns under the condition that the Kevlar content proportion is constant, and the maximum energy absorption can be increased by 24.92%.

## 1. Introduction

Carbon fiber-reinforced polymer (CFRP) composites are characterized by low density, strong design flexibility, and high specific strength and modulus. They are gradually being used in aerospace, automotive, and other fields. However, the high modulus and strength of carbon fiber result in low toughness and poor interlayer performance. Under impact loading, CFRP composites are prone to delamination and structural failure. In contrast to traditional laminated structures, three-dimensional (3D) woven composites incorporate fibers in the thickness direction, which enhance anti-delamination and impact resistance. Studying the impact resistance of 3D woven composites is particularly important for the application of the composite.

Turner et al. [1] investigated the damage mechanisms of orthogonal 3D woven composites under impact loading. Baucom et al. [2] performed drop weight impact tests on two-dimensional (2D) woven composites, 3D woven composites, and biaxial braided composites. The results indicated that the 3D structure exhibited higher impact resistance and greater energy absorption. Bahei-EI-Din et al. [3] examined the deformation and damage mechanism of 2D and 3D braided composites under impact loading and found that significant delamination did not occur in the 3D braided composites. Roberts et al. [4] conducted ballistic target shooting tests on 3D woven plates with various components and found that the type of carbon fiber significantly influenced the critical velocity of the target. Munoz et al. [5] conducted ballistic impact tests on the 3D woven plate with mixed carbon fiber and glass fiber. The results showed that when carbon fiber was used as the rebounding surface, the critical velocity of the target plate exceeded that of glass fiber used as the rebounding surface.

Recent advancements in composite materials have revealed that the strategic incorporation of high-toughness fibers into polymer matrices can dramatically improve impact resistance characteristics. These findings have stimulated significant research efforts focused on developing hybrid composite systems that synergistically combine multiple fiber types to achieve superior mechanical performance [6,7,8]. Luo et al. [9] conducted transverse impact tests on 3D orthogonal hybridized woven composites, showing that no delamination occurred under transverse impact. Nisinini et al. [10] compared the impact resistance of carbon fiber composites and carbon basalt–flax composites, finding that the energy absorption of both composites was similar. However, the carbon basalt–flax composite exhibited less fragment diffusion upon damage. Pandya et al. [11] conducted an experimental study on the impact resistance of four different types of symmetrical hybrid composites, finding that adding a glass fiber layer to the carbon fiber structure could enhance the ballistic limit speed of the target. Muhi et al. [12] analyzed the impact performance of glass fiber and Kevlar fiber hybrid composites and discussed the significant effects of layup sequence and bullet shape on the target’s impact performance. Hazell and Appleby-Thomas [13] conducted ballistic impact tests on symmetrical carbon fiber composites, finding that the impact resistance of the structure was enhanced by adding either Kevlar or aluminum layers. However, adding the Kevlar layer before the carbon fiber layer had minimal effect on ballistic performance. Fujii et al. [14] investigated the impact perforation behavior of CFRP composites, demonstrating that the static tensile fracture energy of the fiber in the back elastic surface of the target correlated with the energy absorption of the target plate.

Finite element (FE) simulation is an effective way to obtain the distribution of stress and damage in structures. Recently, research has gradually developed to investigate the mechanical behavior of 3D braided composites through finite element simulations. Sohail et al. [15] utilized continuous shell elements and the traction separation method to simulate the interlayer behavior, characterizing the impact process of hybrid 3D woven composites. Tan et al. [16] developed a program to automatically construct the finite element model. The accuracy of the model was verified through experiments. Based on the macro model, Xiao et al. [17] established an impact shear model for plain weave glass fiber resin composites, simulating material softening behavior under various loading conditions with four different damage parameters. Based on the experiments, Ha-Minh et al. [18] numerically simulated the static and dynamic mechanical properties of yarn and studied how the deformation rate affected these properties. Yen [19] established a constitutive model for the braided composite laminate and analyzed two matrix-related failure modes. The progressive damage of the laminate under ballistic impact tests was analyzed. Ha-Minh et al. [20] proposed a new tool for generating geometrical models of fabrics in shell or solid elements and investigated the effect of friction on the ballistic impact performance of interlocking structures. It was found that larger coefficients of friction could improve the ballistic performance of fabrics.

With the development of artificial neural networks (ANNs) in artificial intelligence, a large number of scholars have begun to use ANN models to study the high-speed impact problems of composite materials. Compared with the above-mentioned experiments and numerical analysis, the ANN model has the advantages of high efficiency and low cost. Artero-Guerrero et al. [21] used a combination of finite element and ANN models to study the effect of the stacking order of symmetrical 12-layer laminates on high-speed impact performance. Malik et al. [22] used the ANN model to predict the energy absorption of composite laminates under low-velocity impact loads. Albanesi [23] optimized the weight reduction in composite laminated turbine blades by combining genetic algorithms and ANNs.

In this paper, the energy absorption of hybridized 3D woven composites was studied. Relevant parameters for four types of 3D woven composites were obtained based on basic performance tests and representative volume cells. A method based on a macroscopic mesoscale finite element model, experimental verification, and an artificial neural network (ANN) was used to study the energy absorption of 3D woven targets. Based on the finite element calculation results, the ANN was trained to study the influence of different yarn mixing schemes on the energy absorption of the 3D woven target, and the optimal combination of yarns of the 3D woven target under different Kevlar proportions was obtained.

## 2. Materials

**Figure 1:**
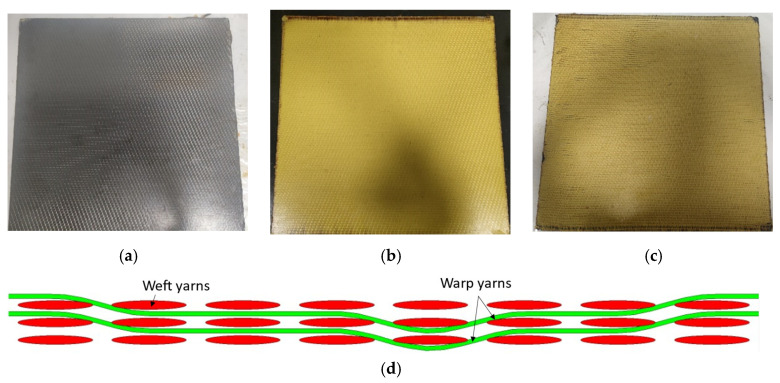
Three-dimensional woven composite panel and weaving structure. (**a**) Carbon structure, (**b**) Kevlar structure, (**c**) hybridized structure, and (**d**) weaving structure.

The hybridized 3D woven composites were designed with four different types of woven panels, each with varying Kevlar content, as shown in Figure 1. Figure 1a–c shows the carbon, Kevlar, and hybrid composite panels. The three-dimensional woven composite’s weaving structure is illustrated in Figure 1d. The structure consists of three weft yarn layers (4 threads/cm) and two warp yarn layers (10 threads/cm).

The materials used in this investigation were TZ800G carbon fiber by Weihai Guangwei Composite Co., Ltd., (Weihai, China), Tapara-na-1670Dtex Kevlar fiber by Tayho Advanced Materials Group Co., Ltd., (Yantai, China), and AG80 epoxy resin by Shanghai Huayi Resin Co., Ltd., (Shanghai, China). The test specimens were fabricated using the RTM process at the Nanjing Fiberglass Research and Design Institute, SINOMA, Nanjing, China. The panels were post-cured in an oven at 60 °C for 8 h. The 500 mm × 500 mm × 2 mm target plates and basic performance test specimens for subsequent testing were obtained via water jet cutting.

## 3. Basic Performance Test

**Figure 2:**
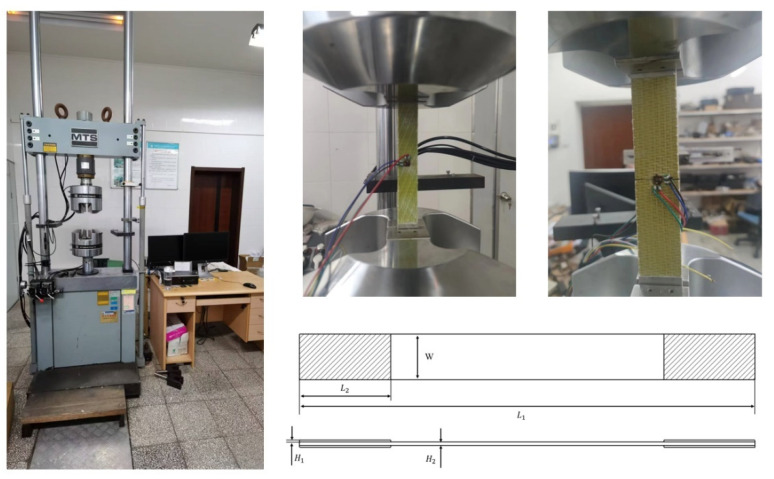
Test equipment and test specimen.

The basic performance tests included warp tensile and compression tests, as well as weft tensile and compression tests. The tests were conducted on the MTS 70.25 testing machine at a constant displacement rate of 2 mm/min. Each group of tests was repeated five times. The testing equipment and specimens are illustrated in Figure 2.

The dimension of the representative volume cells of the woven structure is 8 mm × 20 mm × 2.25 mm. To minimize the impact of the test specimen’s size on the mechanical behavior of the material, the specimen width should contain at least two single cells. The dimensions of the test specimen are presented in Table 1, where *L*_1_, *L*_2_, *W*, *H*_1_, and *H*_2_ are shown in Figure 2.

## 4. Basic Performance Model

### 4.1. Representative Volume Cell

**Figure 3:**
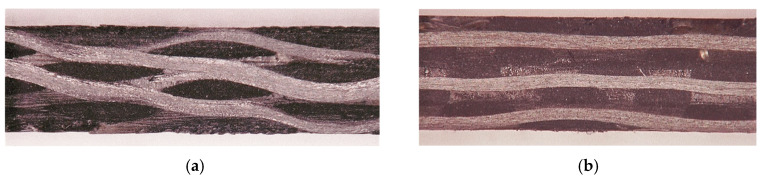
Microscopic photos. (**a**) Cross-sectional view along warp direction. (**b**) Cross-sectional view along weft direction.

Based on the microscopic observations shown in Figure 3, the cell models of four weaving structures were established. The following assumptions were made to establish the representative volume cell (RVC) models [24,25]:

(1) The weft yarns were straight along the thickness direction;
(2) The cross-section of the warp yarns was rectangular, and the cross-section of the weft yarns was hexagonal to include the mutual squeezing effect between yarns;
(3) The weft yarns remained straight in the fabric.

**Figure 4:**
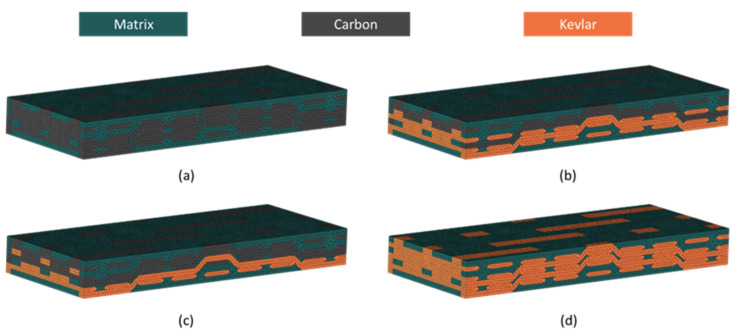
Representative volume cells of the hybridized 3D woven composite. (**a**) C, (**b**) CK, (**c**) KC, (**d**) K.

The representative volume cells of the four types of weaving structures are shown in Figure 4. The model represents weft yarns with hexagonal cross-sections and warp yarns with rectangular cross-sections, with dimensional parameters calculated as described in References [26,27,28]. Based on the Kevlar fiber content variations, the four types of specimens are named C, CK, KC, and K, and shown in Figure 4a–d, respectively. In the finite element (FE) simulations using ABAQUS V2021, the yarn and the matrix were modeled using four-node linear tetrahedral elements (C3D4). This approach simplified the merging of nodes at the contact surfaces of the yarns and the matrix, with total element numbers of 272,952 (yarns) and 177,270 (matrices), respectively. To simulate structural delamination, cohesive elements were inserted between the yarn and the matrix. The total element number of cohesive elements was 64,726.

### 4.2. Progressive Damage

Numerous criteria have been proposed to characterize composite failure and damage [29,30,31]. Among these, the Hashin criterion effectively distinguishes different failure modes of materials. The modified 3D Hashin criterion, represented by Equation (1) to Equation (6) [32], is adopted for yarn’s damage criterion in the present paper.

Fiber tensile failure ($\sigma_{11}>0$) (SDV1):

(1) $${\left(\frac{\sigma_{11}}{X_{t}}\right)}^{2}+{\left(\frac{\frac{\tau_{12}^{2}}{2G_{12}}+\frac{3}{4}\alpha_{s}\tau_{12}^{4}}{\frac{S_{12}^{2}}{2G_{12}}+\frac{3}{4}\alpha_{s}S_{12}^{4}}\right)}^{2}+{\left(\frac{\frac{\tau_{13}^{2}}{2G_{13}}+\frac{3}{4}\alpha_{s}\tau_{13}^{4}}{\frac{S_{13}^{2}}{2G_{13}}+\frac{3}{4}\alpha_{s}S_{13}^{4}}\right)}^{2}=1.$$

Fiber compressive failure ($\sigma_{11}<0$) (SDV2):

(2) $${\left(\frac{\sigma_{11}}{X_{t}}\right)}^{2}+{\left(\frac{\frac{\tau_{12}^{2}}{2G_{12}}+\frac{3}{4}\alpha_{s}\tau_{12}^{4}}{\frac{S_{12}^{2}}{2G_{12}}+\frac{3}{4}\alpha_{s}S_{12}^{4}}\right)}^{2}+{\left(\frac{\frac{\tau_{13}^{2}}{2G_{13}}+\frac{3}{4}\alpha_{s}\tau_{13}^{4}}{\frac{S_{13}^{2}}{2G_{13}}+\frac{3}{4}\alpha_{s}S_{13}^{4}}\right)}^{2}=1.$$

Matrix tensile cracking in direction 2 ($\sigma_{22}>0$) (SDV3):

(3) $${\left(\frac{\sigma_{22}}{Y_{t}}\right)}^{2}+{\left(\frac{\frac{\tau_{12}^{2}}{2G_{12}}+\frac{3}{4}\alpha_{s}\tau_{12}^{4}}{\frac{S_{12}^{2}}{2G_{12}}+\frac{3}{4}\alpha_{s}S_{12}^{4}}\right)}^{2}+{\left(\frac{\tau_{23}}{S_{23}}\right)}^{2}=1.$$

Matrix compressive cracking in direction 2 ($\sigma_{22}<0$) (SDV4):

(4) $${\left(\frac{\sigma_{22}}{Y_{t}}\right)}^{2}+{\left(\frac{\frac{\tau_{12}^{2}}{2G_{12}}+\frac{3}{4}\alpha_{s}\tau_{12}^{4}}{\frac{S_{12}^{2}}{2G_{12}}+\frac{3}{4}\alpha_{s}S_{12}^{4}}\right)}^{2}+{\left(\frac{\tau_{23}}{S_{23}}\right)}^{2}=1.$$

Matrix tensile cracking in direction 3 ($\sigma_{33}>0$) (SDV5):

(5) $${\left(\frac{\sigma_{33}}{Z_{t}}\right)}^{2}+{\left(\frac{\frac{\tau_{13}^{2}}{2G_{13}}+\frac{3}{4}\alpha_{s}\tau_{13}^{4}}{\frac{S_{13}^{2}}{2G_{13}}+\frac{3}{4}\alpha_{s}S_{13}^{4}}\right)}^{2}+{\left(\frac{\tau_{23}}{S_{23}}\right)}^{2}=1.$$

Matrix compressive cracking in direction 3 ($\sigma_{33}<0$) (SDV6):

(6) $${\left(\frac{\sigma_{33}}{Z_{c}}\right)}^{2}+{\left(\frac{\frac{\tau_{13}^{2}}{2G_{13}}+\frac{3}{4}\alpha_{s}\tau_{13}^{4}}{\frac{S_{13}^{2}}{2G_{13}}+\frac{3}{4}\alpha_{s}S_{13}^{4}}\right)}^{2}+{\left(\frac{\tau_{23}}{S_{23}}\right)}^{2}=1,$$

where $\sigma_{11}$, $\sigma_{22}$, $\sigma_{33}$, $\tau_{12}$, $\tau_{13}$, and $\tau_{23}$ are the stress components, and $\alpha_{s}$ is the shear nonlinear factor, set to 2.44 × 10^−8^ MPa [33] in the simulations.

The von Mises stress criterion is used for the damage criterion of the matrix in the representative volume cell:

(7) $${\left(\sigma_{1}-\sigma_{2}\right)}^{2}+{\left(\sigma_{2}-\sigma_{3}\right)}^{2}+{\left(\sigma_{3}-\sigma_{1}\right)}^{2}+6\left(\tau_{12}^{2}+\tau_{23}^{2}+\tau_{31}^{2}\right)=2\sigma_{m}^{2}.$$

Except for carbon fibers, both Kevlar fibers and their matrices are sensitive to the strain rate. As the strain rate increases, both the strength and failure strain of Kevlar fiber rise. As the strength of the matrix increases, the failure strain decreases with the increase in the strain rate.

To characterize the effect of strain rate on Kevlar fibers, the Johnson–Cook constitutive model is used:

(8) $$\sigma_{d}=\sigma_{0}\left[1+CIn\left(\frac{\dot{\varepsilon }}{{\dot{\varepsilon }}_{0}}\right)\right],$$

where $\sigma_{d}$ is the dynamic tensile strength of Kevlar fibers, $\sigma_{0}$ is the quasi-static tensile strength of Kevlar fibers, and $\dot{\varepsilon }$ and $\;{\dot{\varepsilon }}_{0}$ are the strain rate and reference strain rate, respectively. In the numerical simulation, the *C* is the strain rate hardening parameter set to 0.04386 [34].

The strain-rate hardening behavior of the matrix is characterized as follows:

(9) $$\sigma_{d}=\sigma_{0}\left(1+b{\dot{\varepsilon }}^{n}\right),$$

where *b* and *n* are the strain rate hardening parameters, and *b* and *n* of the matrix are set to 1.1 and 0.4478 [35] in numerical simulations.

The material parameters of the yarn and matrix used in the mesoscopic model are listed in Table 2 and Table 3, respectively.

A cohesive element was used to simulate delamination between the yarn and matrix in the structure. When the contact stress reached its maximum, the interface began to delaminate:

(10) $${\left\{\frac{\left\langle t_{n}\right\rangle }{t_{n}^{o}}\right\}}^{2}+{\left\{\frac{t_{s}}{t_{s}^{o}}\right\}}^{2}+{\left\{\frac{t_{t}}{t_{t}^{o}}\right\}}^{2}=1,$$

where $t_{n}$, $t_{s}$, and $t_{t}$ are the normal (along the local three direction) and two shear traction (along the local 1 and 2 direction) stress vectors, and $t_{n}^{max}$, $t_{s}^{max}$ and $t_{t}^{max}$ are the peak values of the contact stress when the separation is either purely normal to the interface or purely in the first or the second shear direction, respectively.

The Benzeggagh–Kenane [36] criterion was used as the failure criterion of cohesive elements:

(11) $$G_{IC}+\left(G_{IIC}-G_{IIIC}\right){\left(\frac{G_{S}}{G_{T}}\right)}^{\eta }=G_{TC},\;G_{S}=G_{II}+G_{III}\;G_{T}=G_{I}+G_{S}$$

where η is the empirical constant, $G_{IC}$, $G_{IIC}$ and $G_{IIIC}$ are the release rate of the type I, type II, and type III strain energy, and $G_{TC}$ is the total critical strain energy release rate. Table 4 shows the epoxy resin cohesive model parameters [7].

The material parameters of the macro-region model were measured by the basic performance test. The results are shown in Table 5.

**Figure 5:**
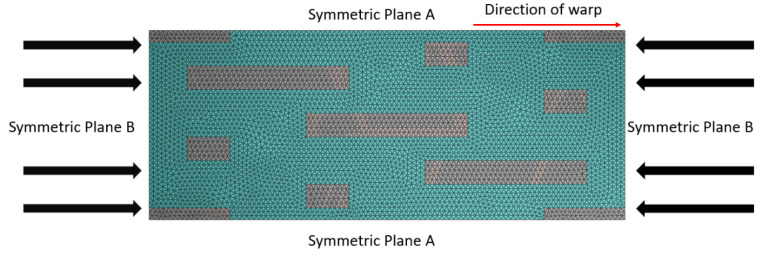
Boundary condition of the representative volume cell.

Due to the periodic nature of the structure, the displacement and stress on relative surfaces of the model must be continuous. Periodic boundary conditions are applied to ensure the continuity of the displacement. Figure 5 illustrates the boundary condition diagram of the model. In the figure, the gray area represents the yarn, the blue area represents the matrix, and the direction of the red arrow indicates the warp direction.

The constraint equation for the periodic boundary conditions were referred to by Xia et al. [37]. The specific equation is as follows:

(12) $$\mu_{i}^{j+}-\mu_{i}^{j-}={\bar{\varepsilon }}_{ik}\left(x_{k}^{j+}-x_{k}^{j-}\right)={\bar{\varepsilon }}_{ik}\Delta x_{k}^{j}$$

where *j+* and *j−* are the positive and negative directions along the $x_{j}$ axis, ${\bar{\varepsilon }}_{ik}$ is the average strain of the RVC, and $x_{k}$ is the coordinates of any point within the RVC.

### 4.3. Comparisons of Experimental Data and Simulations

**Figure 6:**
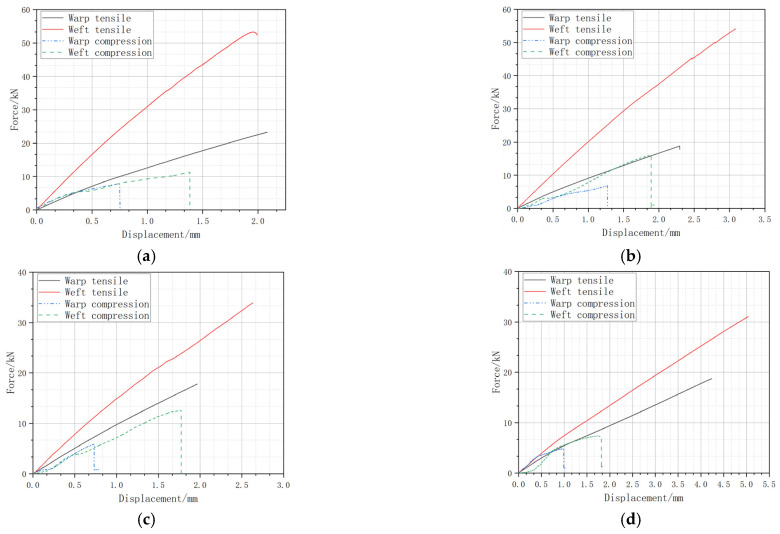
Force–displacement curves obtained from tensile and compressive tests. (**a**) C, (**b**) CK, (**c**) KC, and (**d**) K.

Figure 6 presents test tensile and compressive load–displacement curves. Table 6 and Table 7 compare the weft tensile test data of the four structures with the simulation results. It is seen that the errors are all within 11%, indicating that the model accurately simulates the performance of these structures.

## 5. Ballistic Test

**Figure 7:**
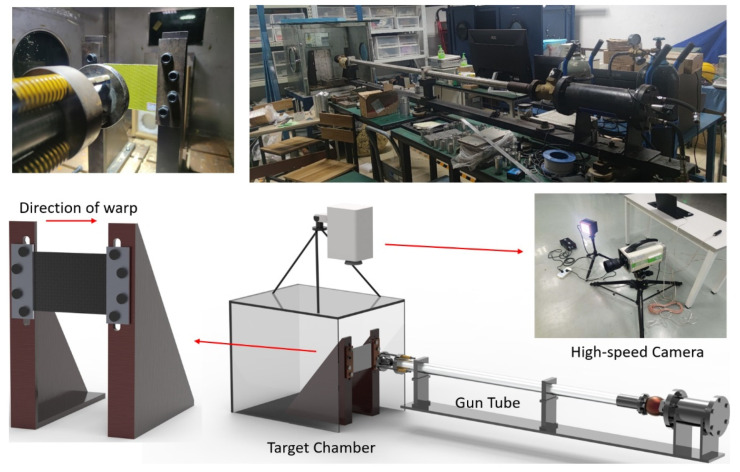
Ballistic target test diagram.

The ballistic test uses air cannon equipment, including a nitrogen cylinder, a gun tube, a target chamber, and a high-speed camera, as shown in Figure 7. In this study, a bullet with a diameter of 6 mm and a mass of 2.3 g is used. The target plate size is 150 mm × 100 mm. In the test, the incident velocity of the projectile is controlled by adjusting the pressure of the gas cylinder, and the high-speed camera is used to record the projectile penetration process and the residual velocity of the projectile.

Table 8 presents the results of the ballistic target test. The ballistic limit velocity was obtained based on the residual velocity by the Lambert–Jonas formula:

(13) $$v_{r}=a(v_{i}^{p}-v_{bl}^{p}{)}^{1/p},$$

where *v_r_* is the residual velocity, *v_i_* is the incident velocity, and *v_bl_* is the ballistic limit velocity. The *a* and *p* are constant.

The ballistic limit velocity of the carbon fiber target plate is 67.57 m/s, and the ballistic limit velocity of the Kevlar fiber target plate is 105.953 m/s. The ballistic limit velocity of hybrid B with higher Kevlar content in the two hybrid target plates is slightly higher (96.115 m/s), and the ballistic limit velocity of hybrid A is 89.296 m/s. When the residual velocity in the table is negative, it means that the bullet did not penetrate the target plate and rebounded.

**Figure 8:**
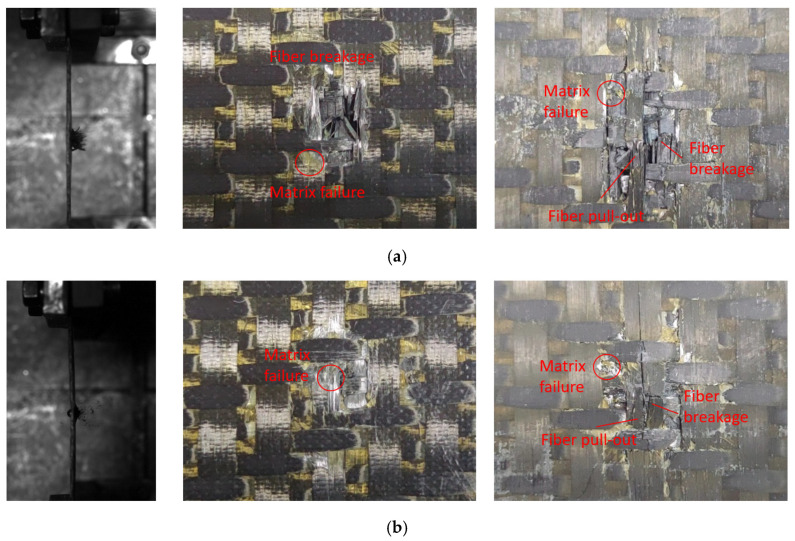
Damages of carbon fiber target plate. (**a**) C-3, (**b**) C-1.

Figure 8 shows the test damage diagrams of the carbon fiber target plates C-1 and C-3. The C-1 target plate was not penetrated. When the bullet struck the target plate, a few matrix fragments flew out from the back, and the impact area showed bending. The C-3 target plate was completely penetrated by the bullet. When penetration occurred, a significant number of fibers and matrix fragments flew out from the back of the target plate during the test, while the overall deformation remained minimal.

Due to being completely penetrated by the bullet, the front of the C-3 target plate exhibited clear fiber breakage and matrix failures, with a distinct crater shape. The primary failure mode on the front surface was fiber shear failure, accompanied by numerous broken fibers and fiber pull-out on the reverse surface. This indicated that the main failure modes of the reverse surface included fiber tensile breakage, fiber pull-out, and resin breakage and peeling.

Different from the C-3 target plate, the C-1 target plate was not penetrated. The front surface of the C-1 target plate clearly showed matrix failure, while the reverse surface was slightly raised. The failure mode resembled that of the C-3 target plate, but the affected area was smaller. In the figure, the red circles represent matrix failure, and the arrows indicate fiber failure.

**Figure 9:**
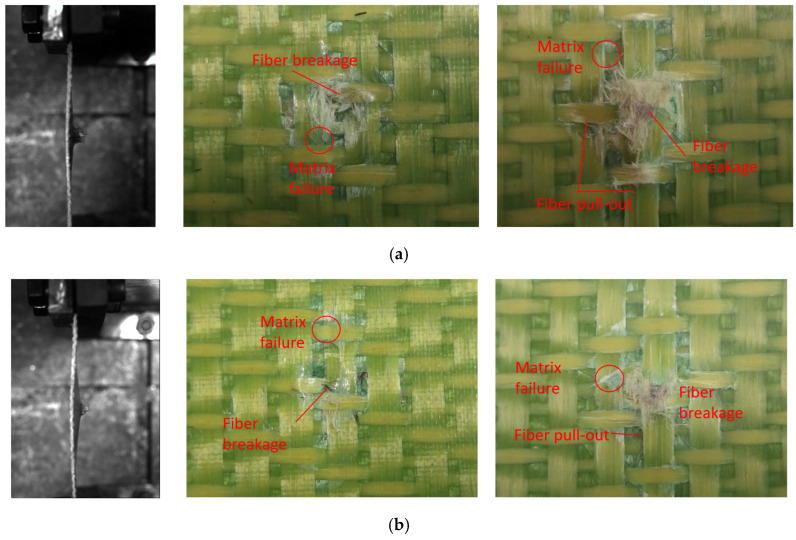
Damage of the Kevlar fiber target plate. (**a**) K-3, (**b**) K-1.

Figure 9 shows the experimental damage diagram of the Kevlar target plate. Compared to the carbon fiber target plate, fewer flying fragments on the reverse surface of the Kevlar target plate were observed during bullet impact tests. However, the Kevlar target plate was noticeably bent, exhibiting significant out-of-plane deformation.

Distinct circular craters appeared on the surface of the K-1 and K-3 target plates, similar to the ones on the carbon fiber target plates. The primary failure modes of the front surface of the Kevlar target plates included fiber fracture and matrix failure, while the reverse surface exhibited tensile fracture, pull-out, and matrix failure.

**Figure 10:**
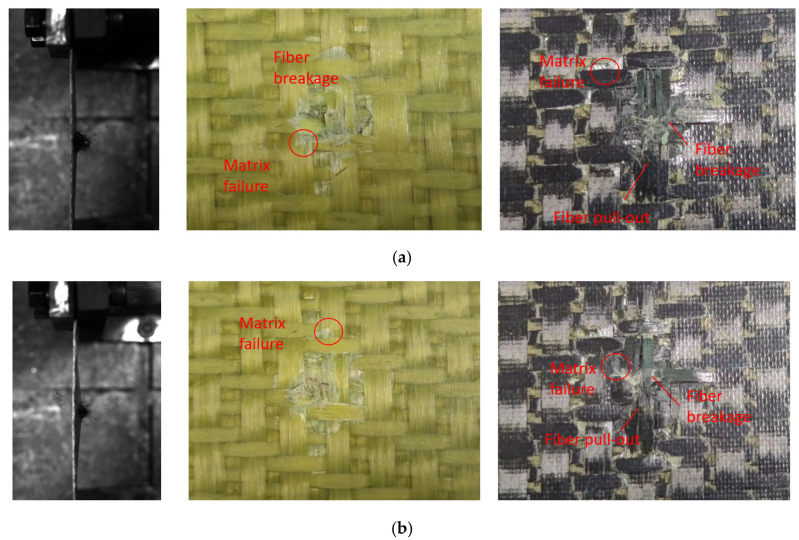
Damage of the hybridized fiber target plate. (**a**) CK-3, (**b**) CK-1.

Figure 10 illustrates the damage diagram of the hybridized fiber target plates. When struck by the bullet, the reverse surface of the target plate resembled that of the carbon fiber target plate, with more matrix fragments. However, the noticeable fiber pull-out phenomenon was not observed. The target plate exhibited less deformation. The CK-1 target plate was not penetrated by the bullet and displayed significant out-of-plane deformation at the point of impact. The failure mode of the front surfaces of the two target plates was similar to that of the Kevlar target plates, exhibiting distinct circular craters. This was accompanied by collective failure and fiber fracture, with the primary failure occurring in the fiber and matrix of the reverse elastic surface. Additionally, a small number of Kevlar fibers appeared at the center of impact.

## 6. Ballistic Target Plate Model

**Figure 11:**
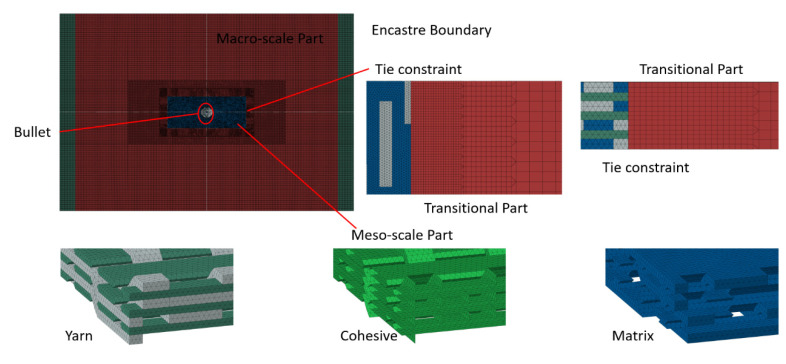
Three-dimensional hybridized woven composite ballistic target model.

The ballistic target plate model was developed using ABAQUS finite element analysis software. The macro-mesoscale modeling technique shown in Figure 11 was used. In the figure, the red region represents the transition zone of the mesh, the blue region represents the matrix in the micro-scale mesh, the dark green represents the weft yarn, the gray represents the warp yarn, and the light green represents the cohesive elements. Mesoscopic modeling at the yarn level was employed only at the impact area, while macroscopic modeling was applied to the rest of the area (the non-impact area). The dimensions of the mesoscopic model were 8 mm × 20 mm × 2.25 mm and modeled by four-node tetrahedral elements (C3D4) with a total number of 2,059,792 elements. The non-impact area employed eight-node hexahedral elements (C3D8R) with a total of 526,756 elements. All impact simulations were performed on a workstation equipped with an Intel^®^ CPU, 16 processors, and 48 GB RAM. To ensure simulation accuracy, mass scaling techniques were not used. Each impact analysis required approximately 13 h of computation time and was time-consuming. The present hybrid composite contained 32,768 (8^5^) possible hybrid configurations. Hence, the artificial neural network (ANN) method was adopted for the primary screening of combinations to reduce the CPU time.

## 7. Artificial Neural Network

Based on MATLAB R2022a software, a multilayer perceptron algorithm (MLP) is written to predict the residual velocity of the target plate with different Kevlar combinations. This approach can effectively overcome the critical limitation of pure FE modeling and simulations.

Although the ANN method can enable rapid performance prediction, it offers less physical interpretability than FE models. To leverage both methods, our workflow employs the trained ANN to predict residual velocities across all design combinations first, then performs FE-assisted micromechanical analysis for critical cases. This hybrid approach effectively integrates FE modeling’s accuracy at key points with ANN’s superior design-space interpolation capability.

**Figure 12:**
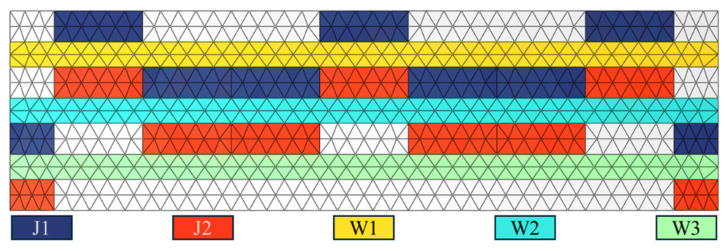
Five groups of warp and weft yarns.

The MLP neural network consists of three parts: the input layer, the hidden layer, and the output layer. Each cell of the 3D weaving structure contains 16 warp yarns and 24 weft yarns. As shown in Figure 12, the warp and weft yarns are divided into five groups. The input layer is the number of Kevlar fibers in each layer, with a total of for layers, and the residual velocity is the output variable. As suggested in the references [21], the hidden layer is one layer.

The present investigation does not consider the arrangement of Kevlar fibers within the same layer. Changes to the Kevlar fibers started from the yarn closest to the impact point and extended outward in the finite element simulations. The input parameters are determined by the Kevlar fiber count per yarn layer. The input combination 87654 corresponds to eight fibers in J1, seven fibers in J2, six fibers in W1, five fibers in W2, and four fibers in W3 regions, where J1, J2, W1, W2, and W3 regions are defined in Figure 12.

## 8. Results and Discussion

### 8.1. Comparisons of Test Data and Simulations

The finite element model simulates the breakdown and non-breakdown of each target. Table 9 compares the test results with the simulations. Simulations of each target plate with two conditions were performed. It was seen that the maximum simulation error was only 6.30% and less than 7%. Additionally, the energy absorbed by the target plate increased with a higher proportion of Kevlar fiber, aligning with the experimental results.

**Figure 13:**
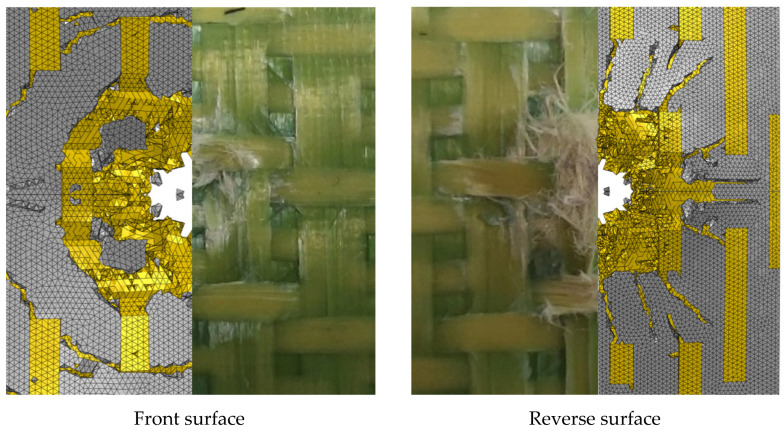
A comparison of the model and test damage.

Figure 13 compares the simulated damage with the test damage of the Kevlar fiber target plate K-3. In the figure, the yellow area represent the yarns and the white area represent matrix. The simulation results align with the actual damage observed on the target plate. Fiber breakage and matrix failures were the primary modes of damage on the front surface. A circular crack in the matrix occurred near the crater. The reverse surface was characterized by fiber pull-out, fiber breakage, and matrix failures.

### 8.2. Analysis of Bullet Impact Process

**Figure 14:**
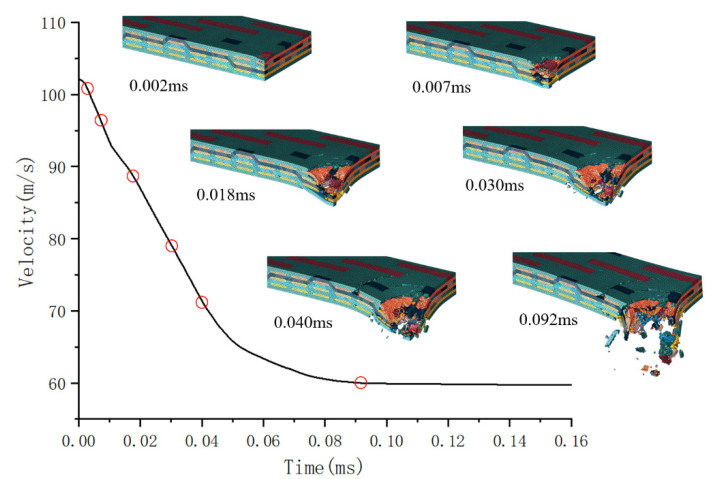
Penetration process on the carbon fiber target plate.

Figure 14 illustrates the penetration process on the carbon fiber target plate at an impact velocity of 102.16 m/s.

At 0.002 ms, the bullet contacts the target plate, creating a circular pit in the contact area. At 0.007 ms, a large area of the matrix is damaged, and the first layer of weft yarn bends due to the impact of the bullet and warp yarn deformation. At 0.018 ms, the upper warp and weft yarns in the structure break due to compression and shear forces. The matrix in the non-contact area cracks, and the yarn at the bottom of the structure is extracted due to the bullet’s impact, while the internal yarn and matrix become visibly separated. The damage degrees of the matrix and yarn increase with the bullet penetrating deeper into the target plate. At 0.040 ms, the warp yarn in the impact zone almost completely fails, the bottom warp yarn breaks, the weft yarn on the inner side breaks due to the bullet’s impact, and the weft yarn close to the outer side bends and twists under the bullet’s action. The matrix in the non-impact zone shows circular cracks. At 0.092 ms, the bullet completely penetrates the target plate, and numerous fibers and matrix fragments are ejected from the back of the target plate.

### 8.3. ANN Prediction Results

Since the efficiency of ANN model learning depends on the randomness of the training dataset, this paper uses random numbers to generate 105 samples. In total, 76 of the 105 samples were used for training, 16 for validation, and 16 for testing.

**Figure 15:**
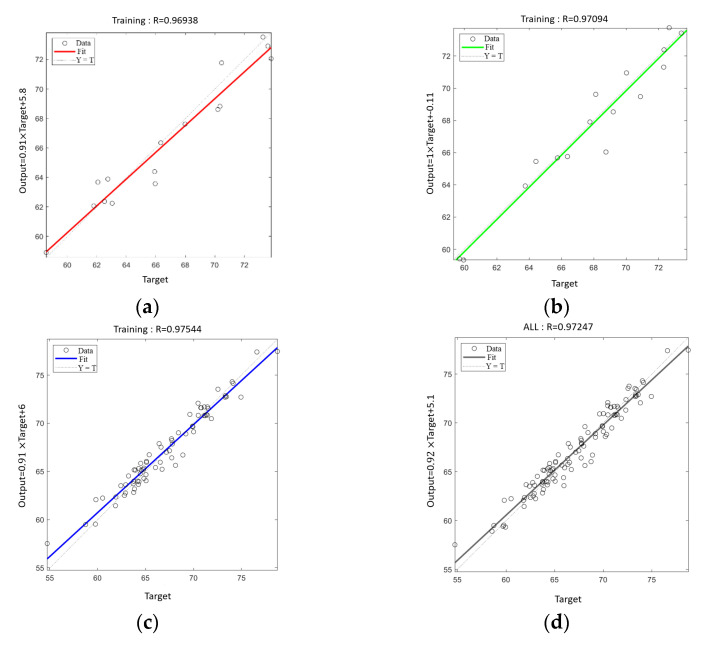
Correlation between the target and predicted values for training. (**a**) Training, (**b**) validation, (**c**) test, and (**d**) total datasets.

Figure 15 shows the correlation between the target and predicted values for training, validation, testing, and total datasets. The X-axis represents target values, while the Y-axis displays corresponding predicted values. The diagonal dashed line indicates perfect prediction (y = x), and the solid line shows the regression curve between predicted and target values. Data points clustering near both the diagonal dashed line and regression curve demonstrate minimal prediction error.

Table 10 shows the residual velocity of the four structures in the simulation and the neural network predictions, indicating the accuracy of the neural network predictions. The mean squared errors (MSE) for the training, validation, and test sets were 1.0039, 1.0661, and 1.4480, respectively.

After the neural network was trained, the results of the four structures in the experiment were compared with the neural network, and the 105 samples provided to the neural network did not include the four results in the experiment. The maximum error between the simulation results and the ANN results of the four target plates was 7.46%. The results show that the ANN model can predict the ballistic limit velocity well and is suitable for obtaining the ballistic limit of different Kevlar hybrid forms.

### 8.4. Location of Kevlar Fiber Hybridization

After completing the training of the artificial intelligence neural network, all possible carbon/Kevlar hybrid configurations within the cell structure were predicted, resulting in a total of 9^5^ = 59,049 combinations. Among these combinations, the target plate with the highest residual velocity was in the all-carbon fiber form. At the same time, the one with the lowest residual velocity was of the all-Kevlar form. This indicates that the incorporation of Kevlar fibers can effectively enhance the impact resistance of the target.

To investigate the effect of each yarn within the structure on impact resistance, several feature combinations from the prediction results will be discussed. Figure 15 illustrates the remaining velocities of specific combinations from the prediction results. In the figure, the *x*-axis represents yarns with Kevlar fibers introduced into the structure, while the *y*-axis represents the remaining velocities. The red area indicates that all other yarns are carbon fibers except for the yarn shown on the *x*-axis, whereas the yellow area indicates that the remaining yarns are Kevlar fibers.

**Figure 16:**
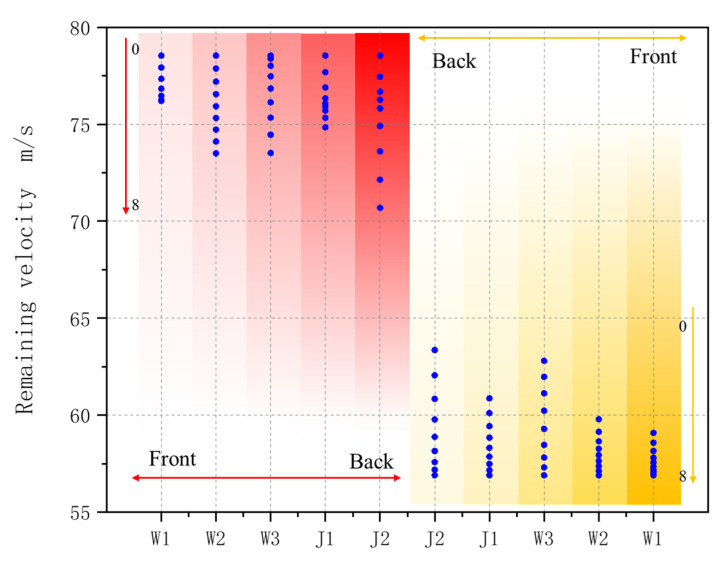
Prediction results of several feature combinations.

As illustrated in Figure 16, as the number of Kevlar fibers increases from 0 to 8, the remaining velocities in each combination decrease. Among the five sets of data in the red area, the J2 group has the smallest residual velocity, which indicates that the hybrid of Kevlar into the J2 warp yarn has the greatest impact effect on the impact resistance of the target plate, followed by W2 and W3. W1, located on the front surface, has the least impact. The J2 group demonstrates the largest residual velocity in the yellow region, followed by W3 and J1. The J2 yarn exhibits the greatest impact on impact resistance in both groups, while the W1 yarn has the least impact.

Under the impact loads, the kinetic energy of the bullet is mainly dissipated by the deformation of the target, fiber stretching, fiber breakage, matrix cracking, delamination, and debonding. In a 3D woven structure, warp threads are interwoven between different layers of weft. Under impact loads, large warp deformation caused by the high toughness of Kevlar fibers can lead to more weft deformation, breakage, and a wider range of matrix cracking. At the same time, the inconsistency of the stiffness of Kevlar fiber and carbon fiber also leads to a further increase in the damaged area.

Kevlar hybridization of warp yarns can effectively improve the energy absorption of the target plate. Compared with the J1 warp, the Kevlar hybridization of the J2 warp yarn in Figure 15 can effectively improve the energy absorption of the target plate. J2 is located on the back of the target plate, intertwined with W2 and W3, and its deformation is greater than that of J1 when impacted. J1 is between W1 and W2, and part of the yarn is on the front side of the target. The deformation of J1 will be affected by the other fibers in the lower layer, and part of the yarn will be cut off by the bullet during impact, resulting in less than the impact of J2.

**Figure 17:**
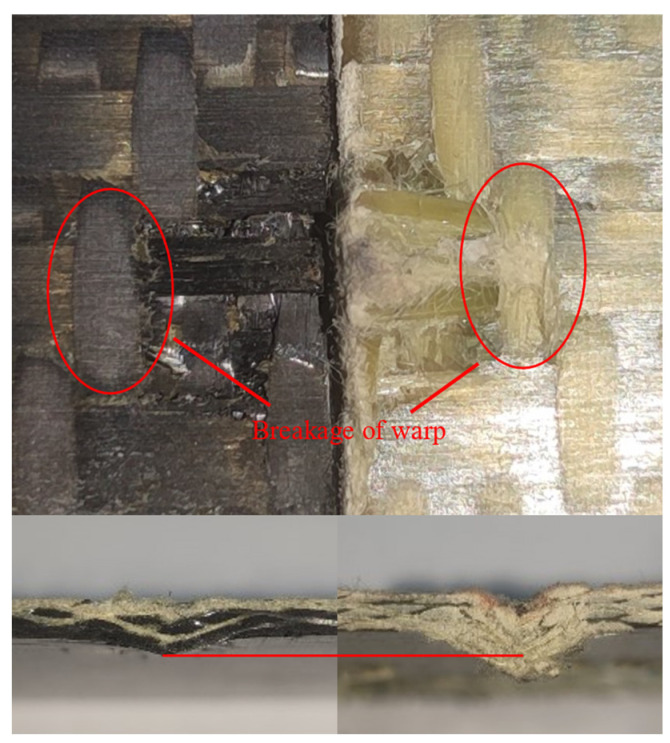
Backside damage diagram of CK and K target plates.

Figure 17 is the impact failure diagram of the target plate with mixed structure and pure Kevlar structure. In the figure, when W3 is made of Kevlar fiber, the damage range on the back side is larger, and the deformation of the target plate at the impact position is greater, which leads to an improvement in the energy absorption of the target plate. At the same time, due to the high toughness of Kevlar, W3 undergoes permanent plastic deformation and causes the surrounding warp to break. When W3 is carbon fiber, it produces a brittle fracture, which has less deformation and less impact on the surrounding warp.

**Figure 18:**
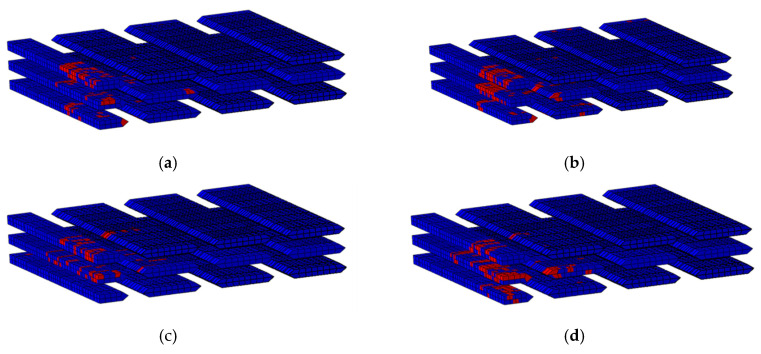
Failure diagram of W3 in four Kevlar hybrid combinations. (**a**) 00080, (**b**) 00000, (**c**) 88888, and (**d**) 88808.

Figure 18 shows the damage of weft yarns in the combination 00000, 00080, 88808, and 88888. In the figure, the red areas represent damaged regions and the blue areas indicate undamaged zones. When W2 is made of Kevlar fiber, the fracture of W3 can be effectively delayed, and the fracture position of W3 is far away from the impact point. A farther fracture position results in a larger damage area and larger matrix damage, as well as more warp deformations and increased energy absorption of the target.

### 8.5. Energy Absorption with Different Kevlar Proportions

**Figure 19:**
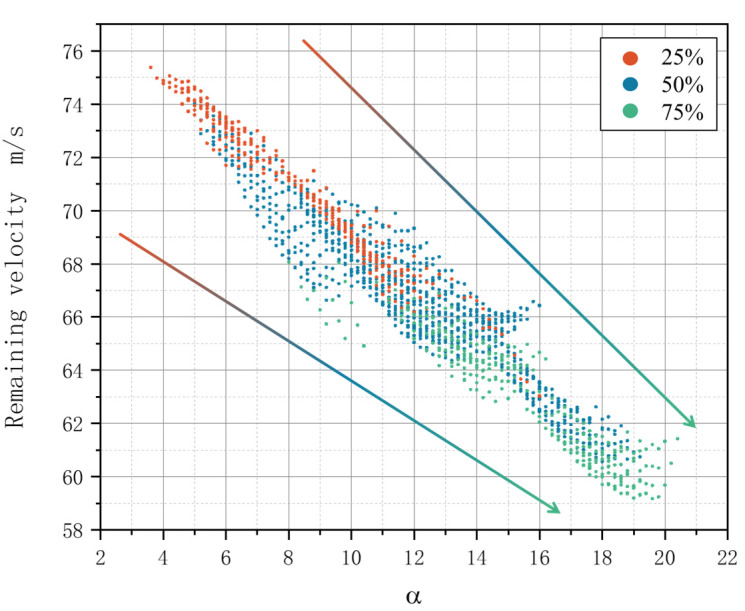
Residual velocity for each combination at different volume ratios.

Figure 19 illustrates the residual velocity for each combination at volume ratios of Kevlar fibers of 25%, 50%, and 75%. In the structure, the volume ratio of a single warp yarn to a single weft yarn is approximately 1:6. Combinations that satisfy the proportion of three Kevlar fibers were selected from all combinations. Among these, 302 groups corresponded to the 25% and 75% combinations, while 909 groups corresponded to the 50% proportion. The x-axis represents the product of the amount of Kevlar in different yarns and its effect on energy absorption, calculated as follows:

(14) $$\alpha =J_{2}\times 1+W_{3}\times 0.8+J_{1}\times 0.6+W_{2}\times 0.4+W_{1}\times 0.2.$$

The arrow of the figure represent the residual velocity of the target plate is inversely proportional to *α*, and the energy absorption of the target plate is directly proportional to *α*.The residual velocity of the target plate in the figure is inversely proportional to *α*, and the energy absorption of the target plate is directly proportional to *α*. The intersection of the three combinations of different Kevlar proportions in Figure 16 indicates that the energy absorption of some targets with low Kevlar proportions will be better than that of some targets with high Kevlar proportions. The energy absorption of the targets will be greatly affected by the Kevlar of yarns at different positions.

Table 11 shows the residual velocity and energy absorption of different combinations under different Kevlar content proportions. When the Kevlar content is 25%, the lowest residual velocity is 63.029 m/s, the energy absorption is 10.246 J, the maximum residual velocity is 75.378 m/s, and the energy absorption is 8.280 J, which is increased by 23.74%. When the Kevlar content is 50%, the lowest residual velocity is 60.457 m/s, the energy absorption is 10.611 J, the maximum residual velocity is 74.133 m/s, and the energy absorption is 8.494 J, which is increased by 24.92%. When the Kevlar content is 75%, the lowest residual velocity is 59.177 m/s, the energy absorption is 10.787 J, the maximum residual velocity is 68.066 m/s, and the energy absorption is 9.487 J, which is increased by 13.70%. The proportion of Kevlar content affects the lower limit of the residual velocity of the target plate, but the hybrid position of different yarns plays a greater role in the impact resistance of the target plate.

## 9. Conclusions

In this paper, the energy absorption of 3D woven composites with varying proportions of Kevlar fibers was studied by a basic performance test, a ballistic impact test, and numerical simulations, and four types of 3D woven composite unit models were established. Based on a combination of macro-mesoscale finite element modeling, experimental verification, and artificial neural networks, a method was used to predict the energy absorption of multiple Kevlar hybrid combinations. The energy absorption characteristics of the three-dimensional woven target structure were revealed under the synergistic effect of Kevlar mixing position and mixing ratio, and the following conclusions may be drawn.

The results from the ballistic target tests and the ballistic target model are consistent regarding the damage manifestation and energy absorption feature, and the maximum error of energy absorption value was only 6.30%. Among the four types of target plates, the 3D woven plate made of pure Kevlar fiber exhibited the highest energy absorption.

Since the pure carbon structure had the highest structural stiffness among the four hybrid forms, the deformation was the smallest under the same energy. The structural stiffness decreased with the increase in the proportion of Kevlar content. This indicates that there is an optimal Kevlar hybrid position and proportion in the study of optimizing the energy absorption characteristics of 3D woven structures to ensure the stiffness index of the structure.

Due to the interweaving between warp and weft yarns in the 3D weaving structure, the position of Kevlar hybridization can result in a considerable enhancement in the energy absorption of the target plate. J2, J1, and W3 have a great influence on the energy absorption of the target plate. Under the impact load, the introduction of more Kevlar fibers into the warp increases its deformation, resulting in a wider range of weft deformation or damage. W3 is located on the back surface of the target plate, and the hybridization of this yarn will lead to increased deformation at the impact point, resulting in a larger area of matrix and warp breakage, thereby improving the energy absorption of the target plate.

Based on the ANN algorithm in this paper, the energy absorption of the target plate can be increased by up to 24.92% by adjusting the hybrid position of Kevlar under consideration of the promiscuous ratio variable.

Future investigations should examine how weaving structures and parameters influence the composite’s impact resistance. Warp yarns interlace with weft yarns across multiple layers, where their bending curvature may significantly influence both in-plane and out-of-plane structural properties. Hence, one may use the combined finite element and ANN technique to predict the energy absorption capacities of various weaving configurations and subsequently optimize the hybrid 3D woven structures.

## Figures and Tables

**Table 1:** Size of the test specimen.

Type	*L*_1_/mm	*L*_2_/mm	*W*/mm	*H*_1_/mm	*H*_2_/mm
Warp tensile	250	50	25	2	1
Weft tensile	280	60	40	2	1
Warp compression	140	13	16	2	1
Weft compression	140	13	20	2	1

**Table 2:** Mechanical properties of yarn.

Properties	Unit	T800	Kevlar
*E*_1_	GPa	247.86	60.31
*E*_2_	GPa	12.88	1.49
*G*_12_	GPa	9.72	6.29
*G*_23_	GPa	5.71	0.60
*μ*_12_		0.35	0.53
*μ*_23_		0.07	0.24
*X_t_*	MPa	3183	1744
*X_c_*	MPa	1328	165
*Y_t_*	MPa	57	58
*Y_c_*	MPa	346	177
*S*	MPa	93	95

**Table 3:** Mechanical properties of the matrix.

Material	Density (kg/m^3^)	Elastic Modulus (GPa)	Poisson’s Ratio	Tensile Strength (MPa)
AG-80	1190	3.5	0.35	112

**Table 4:** Mechanical properties of the cohesive element.

$t_{n}^{max}$ (MPa)	$t_{s}^{max}$ (MPa)	$t_{t}^{max}$ (MPa)	$G_{IC}$ (J/m^2^)	$G_{IIC}$ (J/m^2^)	η
30	80	80	520	970	1.2

**Table 5:** Mechanical properties of the macro-region model.

	Carbon Target	Kevlar Target	Hybridized Target A	Hybridized Target B
*E*_1_/GPa	50.59	25.09	33.01	29.67
*E*_2_/GPa	79.78	25.71	55.20	43.13
*G*_12_/GPa	3.63	2.78	3.38	2.90
*G*_23_/GPa	3.63	2.78	3.38	2.90

**Table 6:** Comparisons of carbon target and Kevlar target test data and simulations.

	Carbon Target			Kevlar Target		
	Experiment	Simulation	Error	Experiment	Simulation	Error
Elastic modulus/GPa	79.78	71.69	10.14	25.71	25.32	1.40
Strength/MPa	628.153	614.48	2.17	553.71	547.60	1.10

**Table 7:** Comparisons of hybridized target A and B test data and simulations.

	Hybridized Target A			Hybridized Target B		
	Experiment	Simulation	Error	Experiment	Simulation	Error
Elastic modulus/GPa	55.19	60.41	9.45	43.13	45.29	5.00%
Strength/MPa	547.37	529.65	3.23	439.39	418.23	4.82%

**Table 8:** Ballistic target test results.

Project	Number	Initial Velocity m/s	Residual Velocity m/s	Absorbed Energy J	Ballistic Limit Velocity m/s
Carbon	C-1	72.14	−12.74	-	67.57
	C-2	97.13	61.66	6.48	
	C-3	102.16	61.69	7.62	
	C-4	115.34	78.66	8.18	
	C-5	120.53	88.32	7.73	
Kevlar	K-1	101.02	−9.58	-	105.953
	K-2	109.01	−10.52	-	
	K-3	119.61	55.44	12.91	
	K-4	130.23	78.43	12.43	
Hybrid A	CK-1	92.38	−13.26	-	89.296
	CK-2	107.56	55.39	9.78	
	CK-3	113.79	70.54	9.17	
	CK-4	124.21	85.477	9.34	
Hybrid B	KC-1	88.89	−12.59	-	96.115
	KC-2	96.29	5.793	10.624	
	KC-3	106.65	50.85	10.106	
	KC-4	113.5	65.6	9.865	
	KC-5	132.5	95	9.136	

**Table 9:** Comparisons of test data and simulations.

Specimen Number	Test			Simulation			Error %
	Initial Velocity m/s	Residual Velocity m/s	Absorbed Energy J	Initial Velocity m/s	Residual Velocity m/s	Absorbed Energy J	
C-1	72.14	−12.74	5.79	72.14	−10.08	5.86	1.20
C-2	102.16	61.69	7.62	102.16	59.71	7.90	3.62
CK-1	92.38	−13.26	9.61	92.38	−8.33	9.69	1.27
CK-2	107.56	55.39	9.78	107.56	50.32	10.39	6.30
KC-1	88.89	−12.59	8.90	88.89	−7.43	9.02	1.33
KC-2	96.29	5.79	10.62	96.29	7.72	10.59	0.28
K-1	101.02	−9.56	11.63	101.02	−4.19	11.72	0.73
K-3	119.61	55.44	12.92	119.61	55.28	12.94	0.16

**Table 10:** Residual velocity of the four structures in the experiment and the neural network predictions.

Number	Simulation			ANN			Error %
	Initial Velocity m/s	Residual Velocity m/s	Energy Absorption J	Initial Velocity m/s	Residual Velocity m/s	Energy Absorption J	
C	113.5	75	8.35	113.5	78.53	7.72	7.46
CK	113.5	72.21	8.81	113.5	73.12	8.65	1.72
KC	113.5	67.94	9.51	113.5	69.23	9.30	2.14
K	113.5	52.36	11.66	113.5	56.90	11.09	4.89

**Table 11:** Residual velocity and energy absorption under different Kevlar hybrid combinations.

Kevlar Content	Combination	Residual Velocity m/s	Energy Absorption J
25%	88103	63.669	10.153
	88310	63.564	10.168
	88004	63.029	10.246
	40600	75.378	8.280
	40510	74.974	8.350
	40501	75.052	8.337
50%	68137	60.457	10.611
	68236	60.563	10.597
	68047	60.658	10.583
	20760	74.032	8.511
	20670	74.133	8.494
	20580	73.971	8.522
75%	48747	59.199	10.784
	47648	59.177	10.787
	48558	59.243	10.778
	00884	68.066	9.487
	00857	67.466	9.580
	00848	68.042	9.490

## Data Availability

The original contributions presented in this study are included in the article.
